# Graph theory reveals dysconnected hubs in 22q11DS and altered nodal efficiency in patients with hallucinations

**DOI:** 10.3389/fnhum.2013.00402

**Published:** 2013-09-05

**Authors:** Marie-Christine Ottet, Marie Schaer, Martin Debbané, Leila Cammoun, Jean-Philippe Thiran, Stephan Eliez

**Affiliations:** ^1^Departement of Psychiatry, Office Médico-Pédagogique (OMP), University of Geneva School of MedicineGeneva, Switzerland; ^2^Signal Processing Laboratory (LTS5), Swiss Federal Institute of Technology (EPFL)Lausanne, Switzerland; ^3^Department of Psychiatry, Stanford Cognitive and System Neuroscience Laboratory, Stanford UniversityPalo Alto, CA, USA; ^4^Adolescence Clinical Psychology Research Unit, University of GenevaGeneva, Switzerland; ^5^Department of Genetic Medicine and Development, University of Geneva School of MedicineGeneva, Switzerland

**Keywords:** DTI, small-world, network, Broca, psychosis, schizophrenia, human connectome, Wernicke

## Abstract

Schizophrenia is postulated to be the prototypical dysconnection disorder, in which hallucinations are the core symptom. Due to high heterogeneity in methodology across studies and the clinical phenotype, it remains unclear whether the structural brain dysconnection is global or focal and if clinical symptoms result from this dysconnection. In the present work, we attempt to clarify this issue by studying a population considered as a homogeneous genetic sub-type of schizophrenia, namely the 22q11.2 deletion syndrome (22q11.2DS). Cerebral MRIs were acquired for 46 patients and 48 age and gender matched controls (aged 6–26, respectively mean age = 15.20 ± 4.53 and 15.28 ± 4.35 years old). Using the Connectome mapper pipeline (connectomics.org) that combines structural and diffusion MRI, we created a whole brain network for each individual. Graph theory was used to quantify the global and local properties of the brain network organization for each participant. A global degree loss of 6% was found in patients' networks along with an increased *Characteristic Path Length*. After identifying and comparing hubs, a significant loss of degree in patients' hubs was found in 58% of the hubs. Based on Allen's brain network model for hallucinations, we explored the association between local efficiency and symptom severity. Negative correlations were found in the Broca's area (*p* < 0.004), the Wernicke area (*p* < 0.023) and a positive correlation was found in the dorsolateral prefrontal cortex (DLPFC) (*p* < 0.014). In line with the dysconnection findings in schizophrenia, our results provide preliminary evidence for a targeted alteration in the brain network hubs' organization in individuals with a genetic risk for schizophrenia. The study of specific disorganization in language, speech and thought regulation networks sharing similar network properties may help to understand their role in the hallucination mechanism.

## Introduction

22q11.2 deletion syndrome (22q11.2DS), also known as velo-cardio-facial syndrome (Shprintzen et al., 1978), is a well-established neurogenetic model for studying the pathogenesis of schizophrenia (Bassett and Chow, 1999). The prevalence rate of the 22q11DS population for developing schizophrenia is about 30%, making it the third highest risk rate after having an affected monozygotic twin (50% risk) or both parents being affected (46% risk) (McGuffin et al., 1995). Furthermore, 30–50% of non-schizophrenic 22q11DS individuals demonstrate sub-threshold symptoms of psychosis (Feinstein et al., 2002). Considering the genetic 22q11.2DS model as a homogenous sub-type may highlight the presence of neurodevelopmental biomarkers underlining the schizophrenic disorders.

As schizophrenia is a heterogeneous disorder previous literature on white matter has revealed highly variable alterations throughout the brain and a few replicated findings (see Fitzsimmons et al., 2013 for a recent review). Due to confounding factors such as duration of illness, medication, age sample or methodology, it remains unclear whether schizophrenia demonstrates localized alterations or a whole brain dysconnection. Graph theory provides promising tools to analyze both whole brain phenomenon using global network measurements, and specific properties using local network measurements (Bassett and Bullmore, 2009; He and Evans, 2010; Rubinov and Sporns, 2010). Few studies in schizophrenia have used the graph theory in structural magnetic resonance imaging. Bassett et al. (2008) characterized the cerebral gray matter volumetric covariation in a large sample (>200) of patients with schizophrenia compared to healthy controls. Although the small-world properties were preserved, a reduced network hierarchy with a loss of hubs was found in individuals with schizophrenia and more specifically in frontal (bilateral dorsolateral prefrontal cortex) areas (Bassett et al., 2008). Graph theory was also used in studies of functional connectivity in schizophrenia. Evidence of global rather than focal functional dysconnectivity grows in schizophrenia literature. Disruption of the small-world properties has been found in patients (Liu et al., 2008) as well as weaker connectivity and lower clustering coefficient (Yu et al., 2012), and hubs alteration (Yu et al., 2012, 2013). The functional connection of several specific regions has also been found such as the temporal lobe, the parietal lobe, the thalamus, hippocampus but more interestingly, functional integration between sub-networks (such as the semantic network, the default network) is impaired (see Zhang et al., 2012; Alexander-Bloch et al., 2013). Using diffusion images for reconstructing brain networks, van den Heuvel et al. (2010) replicated the observation that individuals with schizophrenia have preserved small-world properties and increased path length in frontal, temporal and occipital areas. Reduction of frontal hubs has also been demonstrated, involving the superior frontal and the anterior cingulate (van den Heuvel et al., 2010). Following Latora and Marchiori's conception of global and local efficiency measuring how well information is exchanged over the network (Latora and Marchiori, 2001), Wang et al. (2011) demonstrated that individuals with schizophrenia have a reduced global efficiency [reduced after controlling for the effect of age, gender and brain size (Wang et al., 2011)]. However, it remains unclear whether the network alterations are caused by the emergence of the schizophrenia disorder or if there is a predetermined network configuration that acts as a vulnerability factor for the later development of schizophrenia.

Amongst all the psychotic symptoms in the 22q11DS, hallucinations are often considered the most clinically salient signs of risk for psychosis (Debbané et al., 2006). Moreover, hallucinations constitute a valid early risk indicator for the development of schizophreniform disorders during adulthood (Poulton et al., 2000). As complex cognitive functions rely on a cerebral network involving several regions, (Sporns, 2010; Bassett and Gazzaniga, 2011) dysfunctions such as hallucination may result from abnormal topological connectivity between these areas (Lo et al., 2011). Structural and functional studies in individuals with schizophrenia suggest that several key regions play a role in the apparition of hallucinations and their severity [(see Allen et al., 2008) for a review]. In schizophrenic patients, reduction in the superior temporal gyrus (STG) gray matter volume has been associated with the severity of hallucinations (Flaum et al., 1995; Gaser et al., 2004; Onitsuka et al., 2004). Loss of gray matter volume in Broca's area has also been associated with this symptom (Gaser et al., 2004; Sumich et al., 2005). Several other brain areas, such as the insula (Shapleske et al., 2002), the thalamus (Neckelmann et al., 2006) and the supramarginal gyrus (Gaser et al., 2004), have been associated with the presence of hallucinations but have failed to show consistent volumetric reduction. Two network studies have explored the relationship between clinical symptoms of schizophrenia and brain network properties. Although van den Heuvel et al. (2010) found no significant association between the Positive and Negative Symptoms Scale (PNASS) (Kay et al., 1987) and topological features, Wang et al. (2011) showed a negative correlation between the PANSS (positive, negative and total scores) and global and local efficiency, meaning that the more severe the symptoms, the lower are both the local and the global topological efficiencies. Lower global efficiency and longer path length has also been related to higher score on the negative PANSS scale (Yu et al., 2011a,b).

The purpose of this present work is to study the global and local network features in a population at high genetic risk for schizophrenia (22q11.2DS) by focusing on the hierarchical structure of the brain network (hub topological configuration). Furthermore, we aim to explore the specific relationship between hallucination symptoms and the local efficiency of the related brain areas. According to Allen's model of brain regions involved in hallucinations (Allen et al., 2008), we suggest that the topological connectivity of the following regions—DLPFC, dorsal anterior cingulate, Broca's area, ventral anterior cingulate, orbitofrontal gyrus, and STG—will be associated with the severity of hallucinations in schizophrenia.

## Materials and methods

### Participants

All the participants underwent the same protocol, which included an MRI session for collecting a structural T1-weigthed image and a diffusion image along with an IQ measure with the Wechsler Intelligence Scale for Children-Third Edition revised (Wechsler, 1991) or the Wechsler Adult Intelligence Scale-III for adults (Wechsler, 1997). The participant or their parents signed consent forms containing information about the study and its purpose. The detailed protocol of the study was previously reviewed and accepted by the Institutional Review Board of Geneva University School of Medicine.

### 22q11.2DS group

Forty-six participants with a 22q11.2DS aged between 6 and 26 (mean = 15.20 ± 4.53), (23 males and 22 females) were recruited through parent associations in French speaking European countries. The 22q11.2 deletion was confirmed by a blood sample analyzed with the Quantitative Fluorescent Polymerase Chain Reaction (QF-PCR) performed on the deleted region. The average IQ was of 77.5 ± 16.6. All the patients were assessed by an experienced psychiatrist using the *Brief Psychiatric Rating Scale* (BPRS) (Leucht, 2005), the *Diagnostic Interview for children and adolescents* (DICA) (Reich, 2000) and the *Structured Clinical Interview for DSM-IV AXIS I Disorders* for adults (SCID) (First et al., 1996). Only one patient fulfilled the criteria for schizophrenia. The average BPRS hallucination subscale for the forty-six participants was 1.63 ± 1.12 and among them fourteen individuals reported to have verbal hallucination.

### Control group

The participants from the control group were recruited among the siblings of the patients and in the community. The 48 control participants comprised 25 males and 24 females, aged from 7 to 24 (mean = 15.28 ± 4.35). The average IQ was of 107 ± 18.12. None of the controls had present or past history of psychiatric or neurological disorders.

### MRI characteristics

Using a Siemens Trio 3 Tesla scanner, we acquired a set of two cerebral MRIs for each participant. A T1-weighted sequence with a 3D volumetric pulse was collected using the following sequence: *TR* = 2500 ms, *TE* = 3 ms, flip angle = 8°, acquisition matrix of 256 × 256, field of view = 22 cm, slice thickness = 1.1 mm, 192 slices. The second MRI was a Diffusion Tensor Imaging (DTI) with the following parameters: number of directions = 30, *b* = 1000 s/mm^2^, *TE* = 82 ms, *TR* = [8300–8800] ms, flip angle = 90°, acquisition matrix of 128 × 128, field of view 25.6 cm, slice thickness = 2 mm.

### Image processing

The two acquired scans were processed for each participant using the Human Connectome Mapper (http://connectomics.org, Daducci et al., 2012). The software is a pipeline of several other software programs, combining each dedicated package for the purpose of creating an individual's connectome. For the T1-weigthed image, FreeSurfer software starts to remove all non-brain tissue, segmenting the image in order to extract the white matter, the sub-cortical gray matter volume and the cortical surface (Dale et al., 1999; Fischl et al., 1999). This step is performed using both intensity and continuity data through the whole 3D volume. The surfaces and volumes generated have been validated against histological studies (Rosas et al., 2002). However, these automatic steps need verification and manual correction if necessary. At the end of this process, three-dimensional volumes or surfaces and a cortical segmentation are available For the diffusion images, first we use a correction for the effect of head motion and distortion of eddy currents through an affine alignment using the FLIRT tool of the FSL-FDT software (Jenkinson and Smith, 2001). The realigned images are used to reconstruct the white matter macroscopic bundles using the streamline deterministic tractography of Diffusion Toolkit (http://trackvis.org/). The registration of the T1-weighted onto the diffusion images is an affine transformation using the *intensity-based linear registration* tool FLIRT. When combined, the intersection of the estimated fibers and the segmented regions creates a connectome which is represented by the connection matrix.

### Network measures

Graph theory describes the human connectome as a network of nodes (in our case cortical regions) and edges (in our case white matter bundles) connecting two nodes. This network can be described either with weighted edges (where edges contain the information about the strength of the two nodes) or with binary edges (where only the existence of a link is represented). Therefore, two kinds of measurements are applicable: measurements on a binary network or on a weighted network. Following the purpose of the present study, which is to try to delineate the core organization of individuals with 22q11.2DS, we decided to explore the configuration of the binary network. Furthermore, binary network analyses have the advantage of showing a low variability in the network measures (Cheng et al., 2012).

In the present study, we used the tools for measuring network properties included in the brain connectivity toolbox developed for Matlab (https://sites.google.com/site/bctnet/) (see Rubinov and Sporns, 2010) for the description and mathematical formula of each measure. The first measurement step is the analysis of the global characteristics of the patients' and controls' networks, using the *Characteristic Path Length*, the *Mean Clustering Coefficient*, the *Global Transitivity*, the *Global Efficiency* and the *Global Degree*. In the context of the binary networks, the *Global Degree* represents the total number of edges (existing connections between two nodes) in the network. The *Mean Clustering Coefficient* measures the potential for functional segregation of the network and is calculated as the mean of the *clustering coefficient*, which is the fraction of the number of neighbors of a node that are also neighbors of each other (Watts and Strogatz, 1998). The *Characteristic Path Length* represents the average of the short path lengths of the network. The short path length is the number of edges that have to be crossed to go from one node to another. The *Characteristic Path Length* therefore measures the functional integration potential of a network. The normalized ratio between the *Mean Clustering Coefficient* and the *Characteristic Path Length* of a network gives the *Smallworldness* measure of the network (Watts and Strogatz, 1998; Humphries and Gurney, 2008). The *Smallworldness* measures the optimality between rapid communication throughout the network (functional integration) and the capacity to process locally based information (functional segregation) (Sporns and Honey, 2006). The optimal balance between the *Characteristic Path Length* and the *Mean Clustering Coefficient* can also be estimated by the *Global Efficiency* and the *local efficiency* (Latora and Marchiori, 2001). The *Global* and *local efficiency* measure how efficiently information is exchanged over the network, and respectively play similar roles to the *Characteristic Path Length* and the *clustering coefficient*.

The second analysis focuses on the hub configuration by ranking all of the nodes in the healthy control network on three local network measures, the *local degree*, the *clustering coefficient* and the *betweenness centrality*. The *local degree* measures a node's number of edges or neighbors. The *clustering coefficient* highlights a node's surrounding configurations by analyzing how many of its neighbors are also connected to each other. The *betweenness centrality* measures how many short path lengths pass through the node and is therefore a measure of node influence on the network (Rubinov and Sporns, 2010). By definition, the 20% highest ranking nodes for all 3 values are considered to be the network hubs (Sporns et al., 2007; Sporns, 2010; van den Heuvel et al., 2010; van den Heuvel and Sporns, 2011). The analysis then continues by comparing the hubs and node degrees between the 22q11.2DS group and the healthy control group.

**Figure 1:**
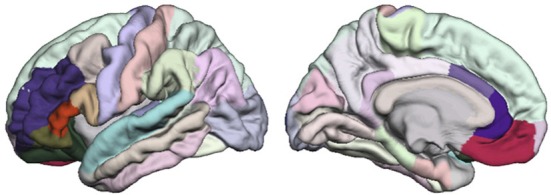
**Among the Desikan parcellation scheme, the regions elected as similar to Allen's model areas are the represented in full color and the remainder parcels are in faded color**. On the lateral view: the superior temporal is cyan, the rostral middle frontal is purple, the lateral orbitofrontal is dark green, the pars orbitalis is khaki, the pars triangularis is dark orange, and the pars opercularis is beige. On the medial view, the medial orbitofrontal is fuchsia, the rostral anterior cingulate is dark purple, and the caudal cingulate is parme.

The third step consists of exploring the relationship between Allen's model's network characteristics (*local efficiency)* and the clinical measures such as the presence and severity of hallucinations (BPRS). According to Allen's model of hallucinations, the DLPFC, dorsal anterior cingulate, Broca's area, ventral anterior cingulate, orbitofrontal gyrus, the supplementary motor area and STG are the brain regions involved in hallucinations. These regions are not represented in the same way in the Freesurfer Desikan parcellation scheme: region that corresponds the most to the DLPFC is the rostral middle frontal parcel; for the superior temporal gyrus it is the superior temporal parcel and the transverse temporal parcel; for the ventral and dorsal anterior cingulated it is respectively the rostral anterior parcel and the caudal anterior cingulate parcel; the inferior frontal gyrus including Broca's area refers to the pars triangularis parcel, the pars opercularis parcel and the pars orbitalis parcel; and the orbito frontal gyrus corresponds to the lateral orbito-frontal parcel and the medial orbito-frontal parcel (see Figure 1). We decided not to include the supplementary motor area, as it is equally spread over three Freesurfer's regions (the caudal middle frontal, the superior frontal and the precentral).

As Latora and Marchiori (2001) demonstrated, the local efficiency measures the functional segregation of one node when this particular node is removed from the sub-network. This measure represents the level of local information-processing. More precisely the local efficiency measures the functional segregation which means the capacity of locally processed information. In the case of hallucination, Allen et al. postulated a neuroanatomical model, composed of several cortical regions where their dysfunctional interplay fosters the hallucination emergence. Therefore, we wanted to see if the local capacity of this sub-network to process the information was related to the emergence and the severity of the hallucination.

In order to analyze the efficiency of communication in Allen's theoretical network, we compared the local efficiency value of each node between patients and healthy participants. Then, in the patients' networks, we analyzed the relationship between each node's efficiency and the BPRS hallucination subscale. For the ten regions, an outlier analysis was applied on the efficiency measurement and the correlation was controlled for age and gender.

### Simulation method

As previous literature in the 22q11.2DS demonstrated that the brains of patients with 22q11.2DS show a 10% volumetric reduction (Eliez et al., 2000; Kates et al., 2001), which has an impact on the number of fibers (~10% less fibers) (Ottet et al., 2013), in the current study we simulated a random 10% reduction on the controls' connectome. This simulation enables a determination of whether the network measures applied for comparing both groups are biased by the reduction of the number of fibers or not. Using Matlab, we randomly subtracted one fiber at a time until 10% of the total number of fibers in the network were removed. This procedure was replicated for each healthy control connectome before applying the global network measurements.

## Results

### Global network measures

All the global network results comparing patients, controls and simulated networks can be found in Table 1.

**Table 1:** **Mean, standard deviation and significant differences in the brain network global measures for participants with 22q11.2DS, healthy participants and simulated networks**.

**Global network measures**	**22q11.2DS**		**Control**		***p***	**Simulated**		***p***
	**Mean**	**Std**	**Mean**	**Std**		**Mean**	**Std**	
Total number of fibers	42087	6608	46764	7343	**0.0005**	42385	5098	0.59
Total number of edges	1269.8	167.5	1354.5	132.4	**0.0038**	1354.5	132.4	**0.0038**
Characteristic path length	2.1652	0.1640	2.1150	0.1069	**0.0402**	2.1150	0.1069	**0.0402**
Mean clustering coefficient	0.7319	0.0295	0.7345	0.0234	0.3224	0.7345	0.0234	0.3224
Global efficiency	0.5238	0.0335	0.5372	0.0221	**0.0117**	0.5372	0.0221	**0.0117**
Smallworldness	1.6837	0.0859	1.68961	0.0783	0.368	1.68961	0.0783	0.368

The first p column from the left reports in bold the significant value (p < 0.05) of the comparison between the 22q11.2DS and control network. The second reports in bold the significant values (p < 0.05) when comparing the 22q11.2DS with the simulated network.

The one tailed *t*-test comparison between the number of fibers contained in the patients' network and the controls' network revealed a significant loss of 10% of fibers (*p* < 0.001). The same test was applied between the patients' and the simulated network revealing that the significance was no longer preserved (*p* = 0.59). Although the simulated network didn't show any difference in the number of fibers compared to the patient group, the number of edges demonstrated a connectivity reduction of 6% (*p* < 0.005). Similarly, the *Global Efficiency* was significantly reduced in the brain network of the patients (*p* = 0.0117). No difference was observed in *the Mean Clustering Coefficient* between the two groups. Although the *Characteristic Path Length* was increased for the patient group (*p* = 0.04), the *Smallworldness* measure did not differ significantly between the two groups (*p* = 0.368).

### Hub analysis

**Figure 2:**
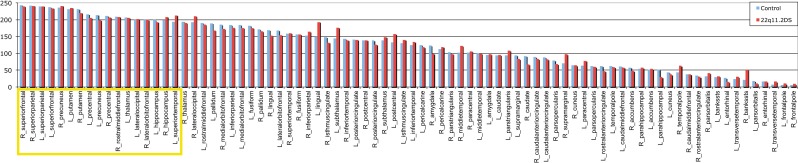
**Final ranking of the 82 gray matter regions in the healthy controls' brain**. The 17 nodes with the highest rank highlighted in yellow, are considered as the connector hubs of the network. In blue is represented the repartition of the final ranking for the control and in red for the 22q11.2DS.

To determine which of the 83 nodes are hubs in the healthy control network, each node was ranked on 3 local network measures: the *local degree*, the *betweenness centrality* and the *clustering coefficient.* According to the literature, hubs are defined as nodes that demonstrate a high degree, a high centrality and a low clustering coefficient. A ranking score was attributed to each of the nodes for the three measures previously discussed and averaged for the 48 healthy controls. The highest connected and the most central node scored 83 and the least connected and least central node scored 1. Inversely the least clustered node scored 83 and the most clustered scored 1. The final classification is the addition of the three scores, in which the top 20% are considered as the connector hubs of the network (see yellow bars in Figure 2). Although the stem node was the highest node on the final classification, we did not consider it as a hub because it is not a gray matter region (therefore all subsequent analyses considered 82 regions and not 83). Twelve out of 17 hubs were represented on both hemispheres in the superior frontal, the hippocampus, the superior parietal, the precuneus, the precentral and the putamen. In the right hemisphere two supplementary hubs are found, the rostral middle frontal and the lateral orbito-frontal. In the left hemisphere, three supplementary hubs were found, the superior temporal the lateral occipital and the thalamus.

### Degree analysis

The results demonstrated that 26 nodes out of 82 (33%) in the patients' networks have a significantly reduced degree after FDR correction (pFDR = 0.0149). The names of the brain regions affected are listed in Table 2. Only one node showed an increased degree in the patients' networks: the right supramarginal region.

**Table 2:** **List of the 26 nodes out of 82 that are significantly different in the patients' network after FDR correction, and indication as being hubs or not**.

**Parcel name**	**Control mean degree**	**Std**	**22q11DS mean degree**	**Std**	**FDR corrected (0.014883)**	**Hub**
R_medialorbitofrontal	16.70	4.36	14.39	4.66	0.00733	No
R_parsopercularis	11.97	3.32	10.04	3.18	0.00245	No
R_rostralmiddlefrontal	21.5	4.52	19.45	3.63	0.00903	Yes
R_superiorfrontal	28.31	3.38	26.30	3.98	0.00492	Yes
R_precentral	23.41	3.96	21.26	3.79	0.00422	Yes
R_caudalantcingulate	10.68	2.41	9.23	2.17	0.00148	No
R_superiorparietal	31	4.35	27.82	6.01	0.00208	Yes
R_inferiorparietal	18.81	5.09	16.71	3.97	0.01451	No
R_precuneus	28.29	3.99	25.45	5.62	0.00290	Yes
R_lateraloccipital	21.70	5.24	18.69	5.74	0.00463	No
R_lingual	18.70	5.27	15.71	5.35	0.00382	No
R_parahippocampal	9.58	3.40	8.06	2.60	0.00874	No
R_inferiortemporal	18.37	5.15	16.10	4.77	0.01488	No
R_hippocampus	21.70	3.36	19.23	3.70	0.00051	Yes
L_parsopercularis	11.91	3.21	9.97	3.44	0.00291	No
L_precentral	24	3.55	20.69	3.36	6.14E-06	Yes
L_superiorparietal	31.91	5.03	28.97	6.19	0.00658	Yes
L_inferiorparietal	21.66	4.27	18.84	4.17	0.00085	No
L_pericalcarine	15.43	4.23	12.89	6.16	0.01064	No
L_lingual	16.52	5.41	13.67	5.06	0.00498	No
L_fusiform	19.18	4.11	14.95	3.34	2.04E-07	No
L_parahippocampal	9.02	3.32	7.52	3.20	0.01432	No
L_middletemporal	16.12	3.96	13.56	4.50	0.00215	No
L_thalamus	24.37	3.00	22.47	3.66	0.00356	Yes
L_caudate	13.81	3.07	11.60	2.86	0.00026	No
L_hippocampus	20.60	3.64	18.02	3.86	0.00062	Yes

**Figure 3:**
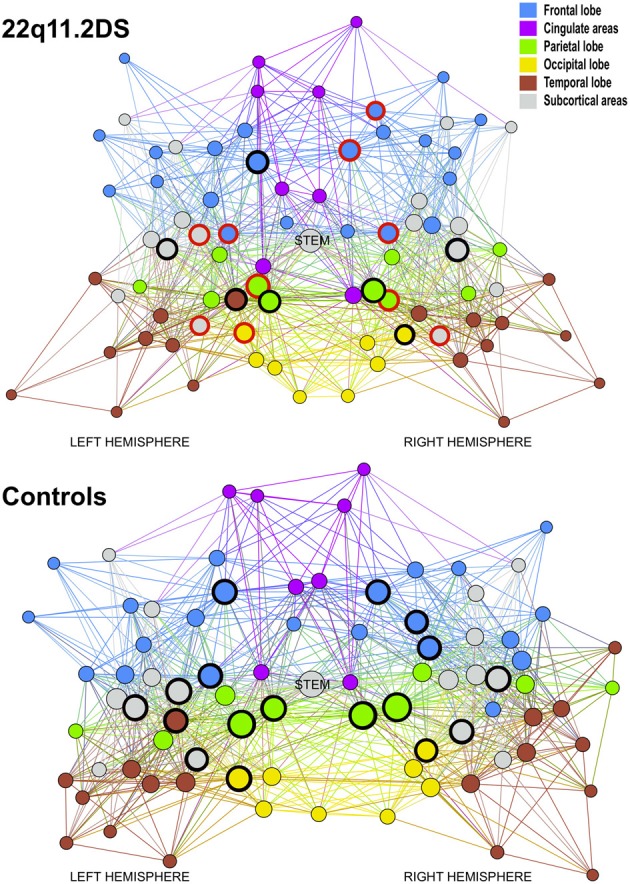
**Graph representation of the mean brain network for patients and controls using Gephi (http://gephi.org/) to produce optimal visualization of all the nodes and connections embedded in the networks**. The circled nodes are the hubs of the network. The red circles are altered hubs and the black circles are preserved hubs. Every nodes contained in the same lobe or cerebral structure has the same color, blue for the node of the frontal lobe, magenta for the cingulate areas, green for the parietal lobe, yellow for the occipital lobe, brown for the temporal lobe and gray for the subcortical areas. The size of the nodes indicates their degree level.

When analyzing how many of the hubs are altered, we noted that 10 out of 17 hubs (58%) were reduced in the patients network (see Figure 3). Conversely only 16 non-hubs out of 65 (25%) were reduced (see Table 2). Therefore, the percentage of affected hubs is more than double that of affected non-hub nodes. The 10 affected hubs are the bilateral hippocampus, superior parietal and precentral regions, right rostral middle frontal, superior frontal, precuneus and left thalamus.

### Efficiency and hallucinations

**Figure 4:**
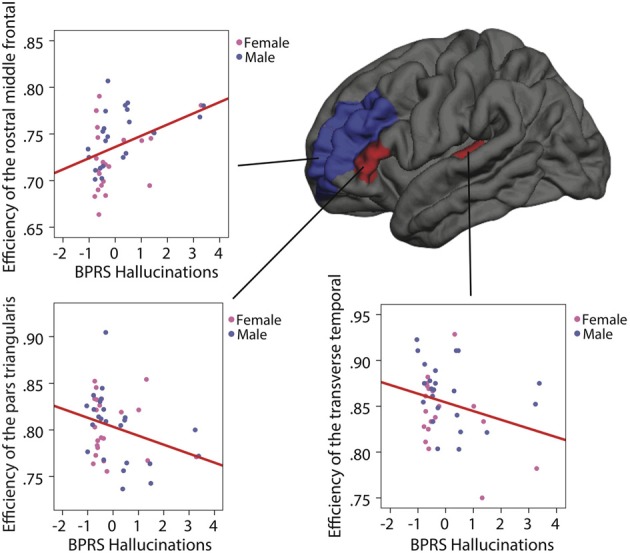
**Correlations between the BPRS hallucination subscale and the network efficiency in individuals with 22q11.2DS after age and gender correction**. On the left hemisphere, the red regions (pars triangularis and transverse temporal) represent a negative correlation and the blue region (rostral middle frontal) represents a positive correlation.

Following Allen's brain network model, the efficiency of ten nodes of the left hemisphere was correlated with the BPRS hallucination subscale. After controlling for age and gender, three of them showed a significant association with the presence and/or severity of the symptoms. Both the pars triangularis parcel and the transverse temporal parcel demonstrated a negative correlation with the symptom's scale (respectively *R* = −0.312, *p* = 0.04, *uncorrected* and *R* = −0.354, *p* = 0.023, *uncorrected*). Inversely, the rostral middle frontal shows a positive correlation with the symptom's scale (*R* = 0.373, *p* = 0.014, *uncorrected*) (see Figure 4). The level of IQ, an additional controlling variable, was introduced, but nevertheless the significance for the three correlations survived.

## Discussion

Using white matter deterministic tractography and gray matter surface-based parcellation to reconstruct the brain connectome, the present work is the first study that analyzes the brain connectome in the 22q11.2DS in the light of the graph theory framework. In line with previous white matter studies in the 22q11DS, we found a 10% reduction in the number of fibers in the brain connectome of patients with 22q11DS (Eliez et al., 2000; Kates et al., 2001; Gothelf et al., 2011; Ottet et al., 2013). We also observed a 6% reduction of connectivity in patients, which means that 6% of the edges of the patients' network were missing. The simulation analysis demonstrated that the initial 10% fiber loss was not the cause of the 6% connectivity loss. This global dysconnection found in our analyses sustains the dysconnection hypothesis in schizophrenia (Friston and Frith, 1995; Stephan et al., 2009).

Despite the observed global disconnection, graph theoretical analysis comparing individuals with a high risk of developing schizophrenia (22q11.2DS) and healthy controls revealed that the smallworldness property of the patients' brain network was still preserved. However, a longer path length, similar to a lower global efficiency, demonstrates that the functional integration [capacity to transmit information more directly, with less interference or attenuation (Latora and Marchiori, 2001)] is reduced in 22q11.2 deletion syndrome. Although the global clustering coefficient is not significantly different from the healthy controls, the small-world brain organization of patients with 22q11.2DS tends to be closer to a regular network organization, in which the functional segregation (capacity to process specialized information, organized in clusters) is preserved but the functional integration is not optimum. Our study is in line with the previous network analysis on non-syndromic schizophrenia patients where a longer *Characteristic Path Length* has been systematically found (van den Heuvel et al., 2010; Zalesky et al., 2010b; Wang et al., 2011).

In the present work, local connectivity analysis allowed localization of the network nodes that were significantly altered in the patients' network. Every lobe and sub-cortical structure had one or more disconnected nodes sustaining the hypothesis that there is a widespread impact on the brain network in schizophrenia (Fornito et al., 2012). However, among the nodes that had a lower connectivity in patients, the proportion of affected hubs compared to non-affected hubs (58%) is interesting. Similarly in schizophrenia, previous findings shows a loss of hub connectivity specifically in frontal lobes (van den Heuvel et al., 2010). Our finding shed light on a possible targeted alteration of cerebral hubs in the 22q11.2DS that may also have some relevance for schizophrenia. Network hubs have special integrative or control functions as their privileged position in the hierarchical organization is postulated to be a key element for large-scale cognitive abilities. Because of their high centrality and influence any perturbation in a hub would heavily impact brain network function (Sporns et al., 2007). The major hub connectivity alteration in individuals with 22q11.2DS may explain their mild cognitive impairments, but also the cognitive collapse seen in schizophrenia during and after their first psychotic episode.

Schizophrenia is a disease with multifactorial etiologies. However, among the multiple causes, genetic factors play an important role (Stephan et al., 2006). Studying brain alterations in populations with high risk or ultra-high risk of developing schizophrenia could break out the predetermining cerebral organization leading to the development of psychosis (Cannon et al., 2007). To the best of our knowledge, no study to date has measured the network properties of white matter connectivity using graph theory in adolescents at risk for schizophrenia. However, a recent study by Shi et al. (2012) demonstrates a significant reduction of global efficiency, an increased global Characteristic Path Length, less hub nodes and lower edge “global efficiency” in neonates' brain networks having a mother with schizophrenia or a schizoaffective disorder. These results suggest that the topological abnormalities in individuals carrying a familial genetic risk for schizophrenia can already be observed a few weeks after birth (Shi et al., 2012). As the 22q11.2 deletion syndrome is commonly considered as a high risk population for schizophrenia (Bassett and Chow, 1999), our study adds some evidence to the hypothesis that early alterations in a cerebral network organization due to genetic factors may partially drive the development of schizophrenia and psychotic symptoms such as hallucinations.

Indeed, the graph theory of large-scale brain networks postulates that cognitive abilities arise from several cerebral regions interacting together (Bressler and Menon, 2010). In our graph network study, we explored the relationship between the alterations of a topological network property and a cognitive dysfunction. Our analyses revealed that the local efficiency value within three parcels of the left hemisphere, namely Broca's area (pars triangularis), Wernicke's area (transverse temporal) and the dorsolateral prefrontal cortex (DLPFC) (rostral middle frontal), correlated significantly with clinical ratings of hallucination severity. Efficiency values in Broca and Wernicke's areas suggested that, as local connections within these parcels decreased, the severity of hallucinatory phenomena increased in our 22q11DS sample. Given the implication of these areas in language production and comprehension, our results suggest that impairments in the different components of language processing in 22q11DS may significantly contribute to the expression of hallucinations. These findings are consistent with previously reported associations between a decrease in verbal IQ and psychotic symptoms (Gothelf et al., 2005; Debbané et al., 2006), and further suggest that local network connectivity in key language areas of the brain contributes to hallucinations in this deletion syndrome (Gothelf et al., 2011).

Hallucination severity further correlated positively with efficiency within the dorsolateral prefrontal parcel. The DLPFC region is one of the most consistently examined regions in MRI studies involving individuals with schizophrenia (Lewis et al., 2004) because of its implications at different levels of impairments, from working memory and executive functions to dopaminergic system malfunction (Tanaka, 2006). Neuromodulation of the DLPFC activity and its role in regulation of thought content is hypothesized to depend on its connectivity patterns with surrounding regions (Arnsten et al., 2012). In this perspective, our results may suggest that an atypically high local efficiency in 22q11DS works against the DLPFC's connectivity with surrounding parcels that modulate its activity. This impairment may thus increase the propensity to experience hallucinations. Future functional MRI studies should examine the connectivity dynamics of the DLPFC more specifically to evaluate its contributions to symptoms such as hallucinations. Overall, our results add important information about the relevance of brain topological network organization to previous longitudinal investigations in 22q11DS that have linked cerebral integrity of gray matter in the DLPFC to the development of psychosis (Gothelf et al., 2005; Schaer et al., 2009; Gothelf et al., 2011). Further research is necessary to understand the maturational dynamics between gray and white matter, and how these may interact to increase the potential for psychotic symptoms in 22q11DS.

## Limitations

In this study, Graph theory principles were applied for the first time on a population with 22q11.2DS bringing new insight on the alterations in their brain organization that could in turn lead to schizophrenia symptoms. Nevertheless, the present work shows some limitations.

Although we choose the recommended ratio between scan timing, voxel size and the number of directions, the same limitations found in every DTI and tractography study remain present. Listed in detail in (Bammer et al., 2003), the major limitations are the absence of *in vitro* validation studies and the distance-related effect of the tractography [see the limitation section in (Ottet et al., 2013)]. This distance related effect biases the number of fibers included in a bundle. Nevertheless, as the present work used unweighted (binary) network analysis, we do not take in consideration the number of fibers, therefore the latter issue is not expected to influence our analyses.

The comparison of brain networks when assessing their properties with graph theory measures, is very sensitive with regard to two constitutive elements, first the choice of the nodes and second the connection density. The first limitation relies on the choice of the cortical parcellation and its scale. Indeed, the choice of a network node is a critical step (He and Evans, 2010). Zalesky et al. (2010a,b) demonstrated the strong influence on network measures of different choices of parcellation scales and diffusion imaging, which impairs the comparability and the constituency across studies (Zalesky et al., 2010a). A wider discrepancy arises when comparing two different parcellation scales (80 vs. 4000 nodes) and/or when comparing two different diffusion imaging techniques [High angular (HARDI) diffusion vs. DTI]. The analyses in our study do not suffer from this issue as we compared our patients' cerebral diffusion image with the same sequence in healthy control cerebral images. Furthermore, for both patients and controls we used the same connectome processing pipeline including the parcellation scheme, which demonstrated a good to excellent test-retest reliability on graph network measures (Owen et al., 2013). Therefore, the comparison between the two populations of the global and local network measures does not suffer from this kind of issue.

Nevertheless, the Desikan parcellation scheme we chose for processing our participants' brain may yield an issue. Although this parcellation is based on the primary and secondary sulci delineation which confers a very high reliability across humans brains (Desikan et al., 2006), the size of the parcels differ importantly. Thus, when delineating the hubs of the network, the very large parcels display a higher degree value, which is one of the three measures used to rank the nodes onto the hub scale. This concern is valid for the two other measures used for delineating the hubs (clustering coefficient and the centrality). However, the hubs we found were highly consistent with previous literature. Indeed, Li et al. (2011) evaluated as the highest reliable hubs the bilateral putamen, bilateral superior frontal and left precuneus among three tractography methods (Li et al., 2011). van den Heuvel and Sporns (2011) found that bilaterally, the precuneus, superior frontal and superior parietal, hippocampus, putamen and thalamus were hubs of structural derived networks (van den Heuvel and Sporns, 2011). Amongst all the hubs delineated in our study, only the bilateral precentral could have been elected because of its large size.

The second limitation concerns the difference in the number of connections that exist between the control and patient networks. This discrepancy may bias the topological measurements and may result in the significant findings. As van Wijk et al. shows in 2010, there is no way to rule out these differences without introducing another bias.

## Conclusion

In the present study, the targeted dysconnectivity of the hubs in a population considered as a model for schizophrenia (22q11.2DS) suggests the existence of an early alteration in the cerebral network organization that is due to genetic factors which may partially drive the development of schizophrenia and psychotic symptoms. Furthermore, altered local efficiency in areas responsible for language processing (Broca and Wernicke) sheds light on the implication of structural network organization in the severity of hallucinations. Further research is needed to understand the interaction between structural networks and psychotic symptoms.

### Conflict of interest statement

The authors declare that the research was conducted in the absence of any commercial or financial relationships that could be construed as a potential conflict of interest.
